# Construction of FeP Hollow Nanoparticles Densely Encapsulated in Carbon Nanosheet Frameworks for Efficient and Durable Electrocatalytic Hydrogen Production

**DOI:** 10.1002/advs.201801490

**Published:** 2018-12-11

**Authors:** Fei‐Xiang Ma, Cheng‐Yan Xu, Fucong Lyu, Bo Song, Shu‐Chao Sun, Yang Yang Li, Jian Lu, Liang Zhen

**Affiliations:** ^1^ State Key Laboratory of Advanced Welding and Joining Harbin Institute of Technology Harbin 150001 China; ^2^ MIIT Key Laboratory of Advanced Structural‐Functional Integration Materials & Green Manufacturing Technology School of Materials Science and Engineering Harbin Institute of Technology Harbin 150001 China; ^3^ Department of Mechanical Engineering City University of Hong Kong Kowloon Hong Kong China; ^4^ Centre for Composite Materials and Structures Harbin Institute of Technology Harbin 150080 China; ^5^ Department of Materials Science and Engineering City University of Hong Kong Kowloon Hong Kong China

**Keywords:** carbon nanosheets, FeP, hollow structures, hydrogen evolution reaction

## Abstract

Developing noble‐metal‐free based electrocatalysts with high activity, good stability, and low cost is critical for large‐scale hydrogen production via water splitting. In this work, hollow FeP nanoparticles densely encapsulated in carbon nanosheet frameworks (donated as hollow FeP/C nanosheets), in situ converted from Fe‐glycolate precursor nanosheets through carbonization and subsequent phosphorization, are designed and synthesized as an advanced electrocatalyst for the hydrogen evolution reaction. FeP hollow nanoparticles are transformed from intermediate Fe_3_O_4_ nanoparticles through the nanoscale Kirkendall effect. The two‐dimensional architecture, densely embedding FeP hollow nanoparticles, provides abundant accessible active sites and short electron and ion pathways. The in situ generated carbon nanosheet frameworks can not only offer a conductive network but also protect the active FeP from oxidation. As a result, hollow FeP/C nanosheets exhibit excellent electrocatalytic performance for the hydrogen evolution reaction in 0.5 m H_2_SO_4_ with a quite low overpotential of 51.1 mV at 10 mA cm^−2^, small Tafel slope of 41.7 mV dec^−1^, and remarkable long‐term stability. The study highlights the in situ synthesis of two‐dimensional metal phosphide/C nanocomposites with highly porous features for advanced energy storage and conversion.

## 1 Introduction

As a renewable and clean energy carrier, molecular hydrogen is expected to be a promising alternative to fossil fuel in the future.^1^ In this regard, sustainable production of hydrogen from electrochemical water‐splitting should be an appealing energy conversion technology due to its low cost and eco‐friendliness.^2^, ^3^ However, the electrochemical water‐splitting demands highly efficient electrocatalysts to decrease the overpotential of hydrogen evolution reaction (HER), a cathodic reaction of full water splitting. As a benchmark and commonly used cathode electrocatalyst in proton exchange membrane (PEM) electrolyzers, platinum (Pt) based catalysts show the highest activity with low overpotential and fast kinetics for HER in acidic electrolyte.^4^, ^5^, ^6^ However, the scarcity and high price of Pt substantially hinder its large‐scale production and applications. Thus, the development of low‐cost, abundant, active, and durable HER catalysts to replace Pt is highly desirable in practical PEM water electrolysis.

Recently, transition metal phosphides, including molybdenum phosphides, cobalt phosphides, nickel phosphides, and iron phosphides, have received intense attention as nonprecious HER electrocatalysts due to their abundant reserves, active properties as well as stability in acids.^7^, ^8^, ^9^, ^10^, ^11^ Especially, FeP, possessing similar catalytic mechanism to highly active [FeFe]‐hydrogenases,^12^, ^13^ is highly promising for large‐scale hydrogen production because iron is the most abundant and inexpensive metallic element. To date, various effective approaches, such as nanostructuring,^14^, ^15^ doping,^16^ compositing,^17^, ^18^ and growing on various 3D conductive substrates to form intergrated electrodes (e.g., carbon cloth),^19^, ^20^, ^21^, ^22^, ^23^ have been developed to enlarge electrochemically active surface areas for enhancing the HER properties of FeP‐based electrocatalysts. Nevertheless, FeP still suffers from poor long‐term stability of HER due to its high oxygen sensitivity during operation. For example, FeP nanowire arrays grown on Ti plate deliver a low overpotential of 55 mV to drive a current density of 10 mA cm^−2^ in 0.5 m H_2_SO_4_. Unfortunately, it exhibits unsatisfied operation stability mainly because of the poor oxidation resistance of nanostructured FeP.^24^ Very recently, researchers found that the HER stability of iron phosphide‐based electrocatalysts can be significantly improved through simply introducing carbon by surface coating owing to the enhanced oxidation resistance. Chung et al. design and fabricate carbon‐shell‐coated FeP nanoparticles deposited on carbon support, which exhibit enhanced HER stability compared to uncoated FeP nanoparticles due to their remarkable oxidation resistance.^25^ However, it is rather difficult to fabricate FeP based 2D nanostructures, essentially because the non‐layer structured FeP phase lacks intrinsic driving forces for anisotropic growth.^26^, ^27^

In the past few years, 2D electrocatalysts have attracted tremendous attention owning to their fantastic features such as superior electron transfer, rapid mass transport, and high percentages of exposed catalytic active surfaces.^28^, ^29^, ^30^ Moreover, the 2D nanostructures tend to form large and continuous conductive membrane on the substrate, which facilitates the electrochemical reactions. Based on the above analysis, 2D FeP nanostructures hybridized with carbon is expected to deliver excellent HER activities. With assembled metal ions and organic ligands into repeating coordination entities, inorganic–organic hybrid materials, such as metal–organic frameworks (MOFs) and metal coordination polymers, are emerging as dual‐functional precursor for in situ construction of carbon frameworks anchoring or encapsulating various metal compound nanoparticles.^31^, ^32^, ^33^, ^34^, ^35^, ^36^ Impressively, a large number of layered inorganic–organic hybrid materials with 2D morphologies have been previously reported, which inspire that we can use the presynthesized 2D inorganic–organic hybrid materials as both template and precursor to fabricate targeted products. Therefore, it is feasible to fabricate 2D FeP/C nanostructures using layered Fe contained metal–organic complexes as precursor.

In this article, we report the design and fabrication of FeP hollow nanoparticles densely encapsulated in 2D carbon nanosheet frameworks (donated as hollow FeP/C nanosheets) through a nanosheet precursor directed topochemical conversion, aiming at boosting both the activity and durability of FeP‐based HER electrocatalysts. Fe‐glycolate, a typical layered metal coordination complex, is selected as the precursor to fabricate 2D FeP/C nanosheets through topochemically carbonization and subsequent gas–solid phosphorization. Interestingly, the hollow FeP nanoparticles are generated via the space‐confined Kirkendall effect. As expected, this integrated 2D mesoporous FeP/C nanosheets can not only offer high conductive network and largely exposed surface sites, but also protect active FeP from oxidation during long‐term electrochemical running. The resultant FeP/C nanosheets exhibit superior electrocatalytic performance in term of a very low overpotential of only 51.1 mV to achieve a current density of 10 mA cm^−2^, a small Tafel slope of 41.7 mV dec^−1^, and remarkable long‐term stability when served as HER catalysts in 0.5 m H_2_SO_4_.

## 2 Results and Discussion

**Scheme 1:**
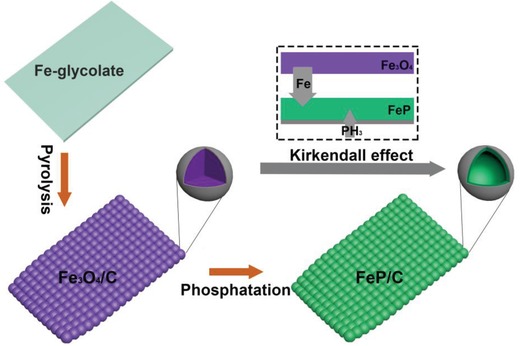
Schematic illustration of fabrication procedures of hollow FeP/C nanosheets.

Hollow FeP/C nanosheets were topochemically derived from Fe‐glycolate precursor through a sequential synthesis process, as shown in **Scheme** **1**. In the first step, well‐dispersed Fe‐glycolate nanosheets, an iron‐contained organic polymer perfectly assembling metal ions with organic ligands, are prepared and served as starting precursor. Subsequently, the as‐prepared Fe‐glycolate nanosheets are topochemically converted to Fe_3_O_4_ nanoparticles encapsulated in carbon framework nanosheets through thermal pyrolysis in inert atmosphere. Finally, hollow FeP/C nanosheets are obtained through a gas–solid phosphorization reaction with the intermediate Fe_3_O_4_/C. During the confined phosphorization reaction with excessive PH_3_, the solid Fe_3_O_4_ nanoparticles in Fe_3_O_4_/C nanosheets can transform to hollow FeP nanoparticles based on nanoscale Kirkendall effect.

Fe‐glycolate precursor nanosheets with high yield and good dispersion were first fabricated through a simple EG‐mediated refluxing process using polyvinylpyrrolidone (PVP) as morphological mediator. X‐ray diffraction (XRD) pattern (Figure S1, Supporting Information) of the precursor sample shows a strong diffraction peak located at 10.9°, suggesting the formation of layered Fe‐glycolate phase.^37^, ^38^ The agitated suspension in ethanol solution shows apparent anisotropic streams (inset in Figure S1, Supporting Information), indicating the anisotropic morphology and high aspect ratio of the precursor sample. The morphology of as‐prepared Fe‐glycolate nanosheets is observed by a scanning electron microscope (SEM) and transmission electron microscope (TEM). The product consists of numerous thin nanosheets with smooth surface and lateral sizes ranging from 1 to 6 µm (Figure S2a,b, Supporting Information). The thickness of Fe‐glycolate nanosheets is estimated to be about 50 nm by atomic force microscope (AFM) topography image (Figure S2c, Supporting Information). In addition, those dispersed precursor nanosheets are generated from the collapse of nanosheet assembled hierarchical structures, which are formed in the initial reaction stage (Figure S3, Supporting Information).

Thermogravimetric analysis (TGA) curve in Figure S4, Supporting Information shows that the Fe‐glycolate precursor would completely decompose before 400 °C in inert atmosphere. Therefore, a moderate calcination temperature of 450 °C is chose to convert Fe‐glycolate precursor to Fe_3_O_4_/C with good crystallinity. In the XRD pattern of calcined sample (Figure S5a, Supporting Information), all the diffraction peaks perfectly match the standard patterns of cubic Fe_3_O_4_ phase (JCPDS card no. 65–3107). Raman spectrum of the calcined sample (Figure S5b, Supporting Information) confirm the presence of amorphous carbon due to the low calcination temperature. The amorphous carbon is originated from the thermolysis of organic group in glycolate precursor. The nanosheet morphology is perfectly preserved after calcination, confirmed by the SEM images (Figure S6, Supporting Information). Through the TEM observation (Figure S7, Supporting Information), carbon covered Fe_3_O_4_ solid nanoparticles with diameter below 20 nm are densely distributed in the nanosheets. Impressively, the nanosheet features are still preserved and numerous pores with sizes of ≈20 nm can be clearly observed after selectively removing Fe_3_O_4_ from Fe_3_O_4_/C using strong acid, suggesting that carbon is the substrate to support and pack Fe_3_O_4_ nanoparticles in the resultant Fe_3_O_4_/C nanosheets (Figure S8, Supporting Information). Based on the above experiments, the calcined product can be identified as Fe_3_O_4_/C nanocomposite, in which the Fe_3_O_4_ nanoparticles are tightly encapsulated in the carbon nanosheet framework. Such carbon nanosheets can provide continuous conductive network, boosting the kinetics of electrochemical reactions.

**Figure 1:**
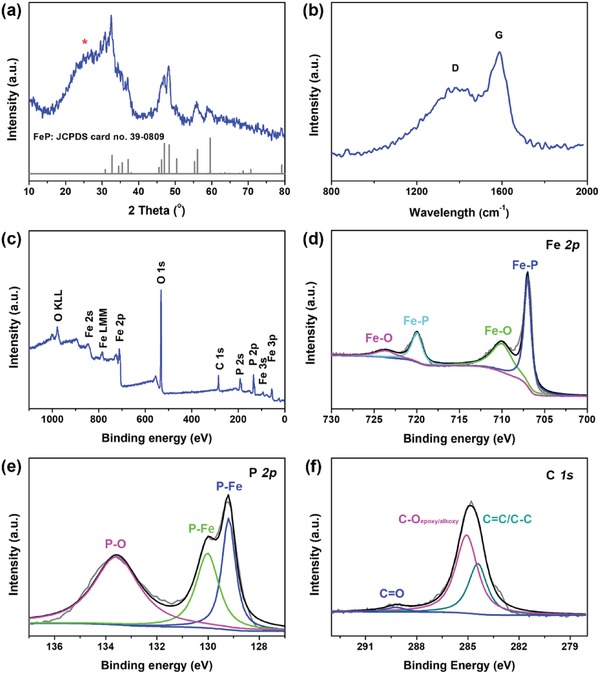
XRD pattern, Raman spectra, and XPS spectra of hollow FeP/C nanosheets. a) XRD pattern with standard crystallographic pattern of FeP (JCPDS No. 39–0809) and b) Raman spectrum of hollow FeP/C nanosheets. XPS survey spectrum c) of hollow FeP/C nanosheets and the corresponding high‐resolution XPS spectra for d) Fe 2p, e) P 2p, and f) C 1s.

Subsequent phosphorization of Fe_3_O_4_/C nanosheets under Ar atmosphere at 350 °C with excessive NaH_2_PO_2_ as the phosphorus source led to disappearance of Fe_3_O_4_ and formation of FeP phase. All the sharp characteristic diffraction peaks of in the XRD pattern of FeP/C nanosheets (**Figure** **1**a) can be well indexed to orthorhombic FeP (JCPDS No. 39–0809),^10^ indicating the complete phosphorization and high crystallinity of the product. Moreover, an additional broad peak located at 26° could be ascribed to the coupled carbon, which is also verified by Raman spectrum (Figure 1b) with obvious D (≈1350 cm^−1^) and G (≈1590 cm^−1^) bands. The pattern of Raman spectrum clearly proves the presentence of amorphous carbon.^39^

To further confirm the formation of FeP/C, X‐ray photoelectron spectroscopy (XPS) analysis was also performed to investigate the compositions and chemical states of the nanosheet product. The survey spectrum (Figure 1c) confirms that the existence of Fe, P, C as well as O elements, which mainly come from the atmospheric contamination and surface oxidation of the sample due to air exposure. The high‐resolution XPS spectrum of Fe 2p (Figure 1d) displays four peaks: two peaks located at 707.1 and 719.7 eV correspond to the binding energy for Fe 2p_3/2_ and Fe 2p_1/2_ in FeP, respectively, and the other two weak peaks at 711.3 and 725.2 eV assigned to oxidized Fe species on the surface of FeP. The P 2p XPS spectrum (Figure 1e) can be deconvolved into three peaks at 133.3, 129.6, and 129.1 eV, respectively. The two high energy peaks located at 129.6 and 129.1 eV are consistent with the binding energy for P 2p_1/2_ and P 2p_3/2_ in FeP.^40^ The C 1s XPS spectrum (Figure 1f) can be deconvolved to three typical peaks at 284.4, 285.1, and 289.1 eV, corresponding to the C = C/C‐C, C‐O, and C = O groups, respectively. These results indicate that the carbon matrix have abundant hydrophilic groups, which would enhance the electrode affinity in the aqueous electrolyte solution.

**Figure 2:**
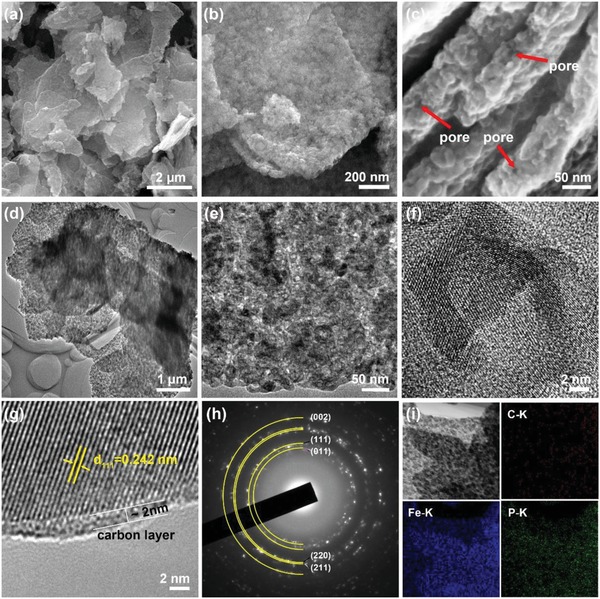
Microstructure characterization of hollow FeP/C nanosheets. a–c) SEM images, d,e) TEM images, f,g) HRTEM image, h) SAED pattern, and i) EDX elemental mapping. The arrows in (c) mark the pores generated from the broken FeP hollow particles in the nanosheets.

SEM images of as‐prepared products show that no structural change or breakage of the 2D structures happen during the phosphorization treatment as shown in **Figure** **2**a,b. There are numerous pores with sizes of several nanometers presented in the nanosheets through carefully observing the lateral and side edges (Figure 2c), indicating the as‐prepared FeP/C nanosheets are highly porous. The energy‐dispersive X‐ray spectrum (EDS) of the as‐synthesized sample shows that the atomic ratio of Fe:P is close to 1:1, further confirming the successful preparation of FeP (Figure S9, Supporting Information). Analysis of Brunauer–Emmett–Teller (BET) adsorption/desorption isotherm (Figure S10a, Supporting Information) suggests that hollow FeP/C nanosheets has a relatively large specific surface area of ≈55.7 m^2^ g^−1^. It should be noted that the calcination temperature of 450 °C is the optimal parameter to obtain the highest surface area of FeP/C nanosheets (Figure S11, Supporting Information). The Barrett−Joyner−Halenda pore‐size distribution curve (Figure S10b, Supporting Information) shows a broad pore diameter ranged from 3 to 20 nm, indicates mesoporous structure of FeP/C nanosheets. Such high surface area with abundant mesopores of as‐prepared FeP/C nanosheets can predictably offer many exposed active sites and abundant transport channels for electrochemical catalytic reactions.

The morphological features and crystal structure of the FeP/C nanosheets were further characterized by TEM analyses. The low‐magnification TEM image in Figure 2d further confirm that the as‐prepared FeP/C nanosheets are highly porous from the apparent contrast. Interestingly, there are numerous hollow particles with diameter of about 20 nm in the nanosheets, as shown in Figure 2e. Unlike MOFs‐derived metal phosphides/carbon hybrids, our FeP nanoparticles are densely distributed in the nanosheet matrix mainly because of the high Fe content in Fe‐glycolate precursor. The high‐resolution TEM image (Figure 2f) shows a single hollow particle with well‐resolved lattice fringes, suggesting that FeP nanoparticles are of high crystalline quality. High‐resolution TEM (HRTEM) image (Figure S12a, Supporting Information) inside the nanosheet clearly shows that crystalline FeP and amorphous carbon co‐existed, confirmed by the fast‐Fourier transform (FFT) patterns (Figure S12b,c, Supporting Information). The formation of FeP hollow nanoparticles from solid Fe_3_O_4_ nanoparticles is attributable to the Kirkendall effect confined in the carbon frameworks.^41^, ^42^, ^43^ Specifically, PH_3_ gas released from NaH_2_PO_2_ can penetrate into the porous Fe_3_O_4_/C nanosheets and simultaneously react with Fe_3_O_4_, forming FeP layers on the surface of Fe_3_O_4_ nanoparticles. As the Fe_3_O_4_ nanoparticles are tightly bound with the carbon nanosheet matrix and the outward diffusion of iron ions is faster than the inward diffusion of phosphorus ions during the phosphorization, the hollow interiors would be created due to the nanoconfined Kirkendall effect. As a result, the precursor Fe_3_O_4_ particles can be converted to FeP hollow particles. Moreover, it is difficult to observe the core‐shell intermediate between Fe_3_O_4_ and FeP, mainly because of the quick completion of diffusion reaction at high phosphorization temperature.^42^

HRTEM image (Figure 2g) taken from the edge of FeP/C nanosheets verifies the existence of iron phosphide, where the FeP (111) crystal plane is clearly seen with a lattice spacing of 0.242 nm. Moreover, FeP nanoparticle is intimately covered by an amorphous carbon layer (marked in Figure 2g) with thickness of ≈2 nm. Selected‐area electron diffraction (SAED) pattern in Figure 2h shows many concentric rings, suggesting a polycrystalline characteristic of as‐prepared FeP/C nanosheets. The related bright discrete diffraction rings can be perfectly indexed to the (011), (111), (220), (211), and (002) planes of orthorhombic FeP, consistent with the XRD result. The corresponding EDX elemental mapping (Figure 2i) of Fe, P, and C for FeP/C nanosheets clearly confirms the homogeneous distribution of Fe, P, and C within the FeP/C nanosheets. Based on these results, hollow FeP/C nanosheets, composed of FeP hollow particles tightly and uniformly encapsulated in amorphous carbon matrix, are successfully prepared through topochemical phosphorization treatment.

**Figure 3:**
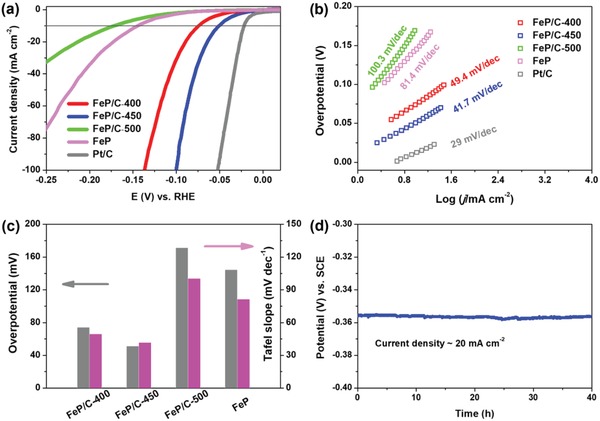
HER performance of hollow FeP/C nanosheets in 0.5 m H_2_SO_4_. a) Polarization curves, b) the corresponding Tafel slope plots, c) overpotentials at a current density of 10 mA cm^−1^ and Tafel slopes of various catalysts (FeP/C, FeP/C‐400, FeP/C‐500, FeP) at a scan rate of 5 mV s^−1^. The LSV curve of Pt/C was also included for comparison and FeP nanosheets were obtained through direct phosphorization with Fe‐glycolate precursor. d) Potential change of FeP/C nanosheets at a constant current density of 20 mA cm^−2^.

The electrocatalytic activity of FeP/C nanosheets toward HER in acidic solution was evaluated using a typical three‐electrode system. All the collected data was corrected for background current and *iR* loss. For comparison, we also examine the catalytic performance of FeP/C nanosheets obtained at different temperatures and phosphorization conditions, which are marked as FeP/C‐400, FeP/C‐450, FeP/C‐500, and FeP to denote the different preparation conditions. Moreover, the electrocatalytic properties of commercially available benchmark Pt/C was measured under identical testing conditions. **Figure** **3**a shows the polarization curves of FeP/C‐400, FeP/C‐450, FeP/C‐500, FeP, and Pt/C in 0.5 m H_2_SO_4_. The hollow FeP/C‐450 nanosheets present a quite low overpotential of 51.1 mV to drive a current density of 10 mA cm^−2^, much better than other FeP based catalysts, including FeP/C‐400 (74.2 mV), FeP/C‐500 (171.7 mV), and FeP (144.7 mV). The overpotential of hollow FeP/C‐450 nanosheets is also close to the catalytic activity of commercial Pt/C with overpotential of 21.9 mV. More impressively, even at a high current density of 100 mA cm^−2^, the overpotential is as low as 100.5 mV for hollow FeP/C‐450 nanosheets. The Tafel slope is an inherent parameter to probe the kinetic behavior and mechanism of HER. The linear portions of the Tafel slopes are fitted with the equation, η = *b*log*j* + *a* (where η is the overpotential, *j* is the current density, *b* is the Tafel slope, and *a* is Tafel constant), as depicted in Figure 3b. The Tafel slope for Pt/C is about 29 mV dec^−1^, which is consistent with previous literature. The Tafel slope for hollow FeP/C‐450 nanosheets is calculated to be 41.7 mV dec^−1^, which is much smaller than that of hollow FeP/C‐400 (49.4 mV dec^−1^), hollow FeP/C‐500 (100.3 mV dec^−1^), and FeP (81.4 mV dec^−1^), indicating the excellent kinetics of the as‐prepared hollow FeP/C‐450 nanosheets. The Tafel slope for hollow FeP/C nanosheets suggests that the HER in acidic electrolyte proceeds by a Volmer–Heyrovsky mechanism, where an adsorbed hydrogen atom at a catalytic interface reacts with a proton electrochemically to produce H_2_. The HER activity of hollow FeP/C‐450 nanosheets was highlighted again for both overpotentials and Tafel slope in Figure 3c.

The long‐term durability of the targeted electrocatalysts, which is essential for practical catalysts, was evaluated by continuous linear sweep voltammetry scans with a sweep rate of 50 mV s^−1^. In general, FeP suffers from unsatisfied electrochemical stability to catalyze HER, mainly because of its high oxygen sensitivity and easy oxidation during operation. In our case, the in situ generated and tightly bound carbon is proposed to serve as the protective layer of the oxygen‐sensitive FeP from oxidation, which can greatly improve the HER stability of FeP. As can be seen, in acidic condition, the polarization curve of FeP/C‐450 hollow nanosheets show no apparent activity loss after 3000 potential cycles, indicating the excellent durability of FeP/C‐450 (Figure S13, Supporting Information). The morphology of FeP/C‐450 hollow nanosheets (Figure S14, Supporting Information) are well retained after the durability testing, again coinciding with the good stability in acidic solution. Moreover, the durability of other electrocatalysts including FeP/C‐400, FeP/C‐450, FeP, and Pt/C was also evaluated (Figure S15, Supporting Information). As predicted, the other hollow FeP/C nanosheets (FeP/C‐400 and FeP/C‐500) also exhibit relatively stable HER properties, which can be ascribed to the protection of in situ generated carbon matrix. However, the HER activity of bare FeP without protection by carbon framework and commercial Pt/C decreased dramatically. Chronopotentiometry is also performed to evaluate the electrocatalytic stability of the targeted catalysts. Figure 3d shows that FeP/C‐450 nanosheets exhibit extreme stability for HER at 20 mA cm^−2^ during 40 h electrolysis process, which is superior to most of ever reported FeP based HER electrocatalysts. The stability test of other electrocatalysts is also performed (Figure S16, Supporting Information). Similar with FeP/C‐450 with carbon protection, both FeP/C‐400 and FeP/C‐500 also exhibit stable activity with ingorable potential shifting. And the bare FeP and Pt/C deliver poor HER stability with significant potential loss. To our best knowledge, the catalytic activity of hollow FeP/C‐450 nanosheets is comparable to that of the FeP nanostructures in situ grown on conductive substrates^20^, ^21^, ^22^, ^23^ with large geometric areas and among the best FeP‐based electrocatalysts, drop‐cast on the flat glassy carbon electrodes, including Fe@FeP/CNT,^44^ FeP/C,^25^, ^45^, ^46^ FeP/graphene,^17^, ^21^, ^40^ and even exceed most of other non‐precious HER electrocatalysts ever reported thus far, such as CoP,^9^ MoP,^47^ Ni_2_P,^8^ Mo_2_C,^34^ MoO_2_,^48^ and CoS_2_@MoS_2_/RGO^49^ (Table S1, Supporting Information).

**Figure 4:**
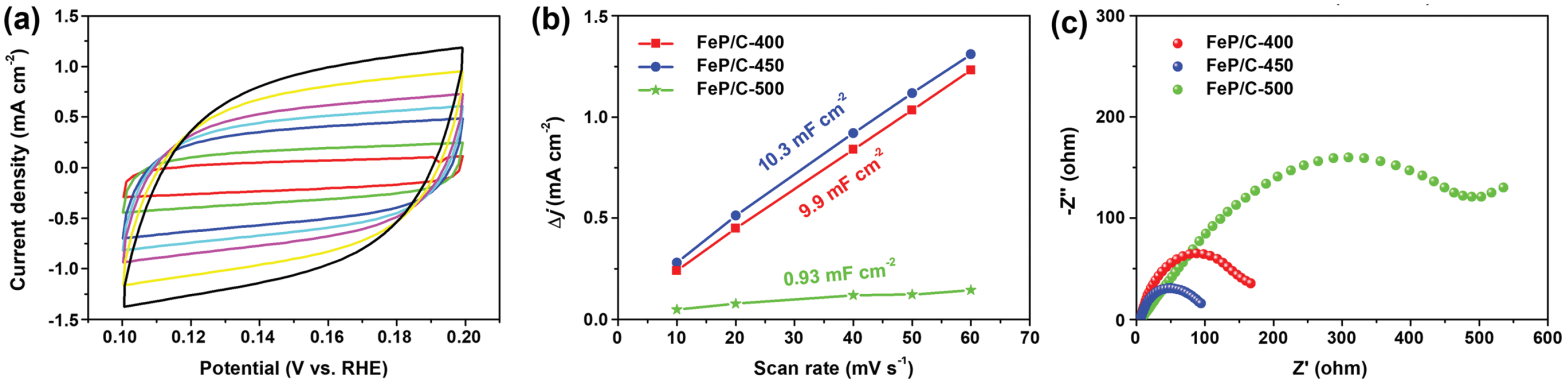
Electrochemical surface area and EIS analysis of hollow FeP/C nanosheets. a) Cyclic voltammetries performed at various scan rates for FeP/‐450 nanosheets. b) Capacitive current plotted against the scan rate at 0.15 V versus RHE and c) Nyquist plots at an overpotential of 40 mV of the as‐synthesized FeP/C catalysts in 0.5 m H_2_SO_4_.

The aforementioned experimental results reveal that the calcination temperature for obtaining Fe_3_O_4_/C is a decisive factor for the electrocatalytic properties of FeP/C nanosheets, suggesting that engineering of porosity and nanoparticular size as well as the carbon crystallinity of FeP/C‐450 nanosheets is important to achieve optimized electrocatalytic properties. The double‐layer capacitances (*C*_dl_) of FeP/C‐450 based electrocatalyst, which is proportional to electrochemically active surface area between the electrocatalysts and electrolyte, can be calculated by the cyclic voltammetry at different scan rates in potential range of 0.1 to 0.2 V versus reversible hydrogen electrode (RHE; **Figure** **4**a). The *C*_dl_ value of the hollow FeP/C‐450 nanosheets (Figure 4b) is as high as 10.3 mF cm^−2^, surpassing those of FeP/C‐400 (9.9 mF cm^−2^) and FeP/C‐500 (0.93 mF cm^−2^) (the values are calculated from Figure S17, Supporting Information). Therefore, the superior HER performance of FeP/C‐450 among all of the hollow FeP/C nanosheets can be acribed to the enlarged electrochemical surface area. Moreover, the electrochemical impedance spectroscopy (EIS) was also performed to probe the interfacial behavior of the resultant FeP/C electrocatalysts, as shown in Figure 4c. It can be seen that the hollow FeP/C‐450 nanosheets show a smallest charge transfer resistance (*R*_ct_) than that of the resultant FeP/C‐400 and FeP/C‐500, revealing fast electron‐transfer kinetics toward HER in the interface between FeP/C nanosheets and electrolyte. Compared with other FeP/C nanosheets, the smaller charge transfer resistance of the FeP/C‐450 nanosheets could be associated with their relatively large surface area.

The outstanding HER electrocatalytic activity of hollow FeP/C‐450 nanosheets can be mainly attributed to the following reasons. First, the high surface area, a highly porous 2D texture, and hollow FeP nanoparticles enable more accessible catalytic sites in hollow FeP/C nanosheets.^50^ Second, the in situ coated and continuous carbon matrix in the FeP/C nanosheets can greatly enhance the electronic conductivity, avoid aggregation of FeP nanoparticles and provide 2D conductive network, all of which would boost the HER kinetics.^51^ Third, the as‐prepared FeP/C nanosheets can easily form highly conductive thin film on the glassy carbon electrode, greatly reducing the resistance of electron and ion transport during electrochemical reaction. More importantly, the coated carbon can also protect active FeP nanoparticles from oxidation, guaranteeing the highly stable HER performance during long‐term operation.

## 3 Conclusions

In summary, hollow FeP/C nanosheets, in which FeP hollow nanoparticles are uniformly and densely encapsulated in the continuous carbon nanosheet matrix, are successfully fabricated using presynthesized Fe‐glycolate nanosheets as precursor via two‐step topochemical routes. The 2D architecture, densely encapsulating FeP hollow nanoparticles, provides abundant accessible active sites and short electron and ion pathways. The in situ generated carbon nanosheet frameworks can not only greatly enhance the electrical conductivity but also protect the active FeP from oxidation. All the structural merits enable the hollow FeP/C nanosheets as efficient and durable electrocatalysts. As a result, the as‐prepared hollow FeP/C nanosheets exhibit outstanding HER performance with low overpotential (51.1 mV at 10 mA cm^−1^), small Tafel slope (41.7 mV dec^−1^), and long‐term stability operated over 40 h. Our work can be extended to the design and synthesis of highly porous metal‐phosphide/C nanocomposites topochemically converted from presynthesized metal–organic complexes with well‐defined morphologies such as metal–organic frameworks and metal–organic coordination polymer.

## 4 Experimental Section

*Materials synthesis*: FeCl_3_·6H_2_O, sodium acetate (NaAc), polyvinylpyrrolidone (PVP, K‐30), and ethylene glycol (EG) were purchased from Sinopharm Chemical Reagent Co., Ltd, and used without further purification. In a typical synthesis of well‐dispersed Fe‐glycolate precursor nanosheets, 12 mmol of NaAc (≈0.984 g) and 2 g of PVP were dissolved in 40 mL EG in a 100 mL three‐necked flask. Then, the reaction mixture was heated to 185 °C at an approximate ramping rate of 15 °C min^−1^. After the reaction temperature reached 185 °C, 2 mL FeCl_3_·6H_2_O EG solution (1 m) was quickly injected into the flask and allowed to react for 2 h under violent stirring. Finally, the product was collected by centrifugation and followed by repeated washing with absolute ethanol for several times, and then dried overnight in an oven at 60 °C. Finally, the as‐prepared precursors were calcined in Ar gas flow at 450 °C for 2 h at a ramping rate of 1 °C min^−1^ to obtain Fe_3_O_4_/C product. In order to fabricate hollow FeP@C nanosheets, 30 mg of Fe_3_O_4_/C sample and 600 mg of NaH_2_PO_2_ were put into a furnace with NaH_2_PO_2_ at the upstream. Then the furnace was programmed at 350 °C with the heating rate of 2 °C min^−1^ and kept for 2 h under an Ar gas flow. After the phosphorization treatment, the furnace was naturally cooled. For the synthesis of hollow FeP/C‐400 and hollow FeP/C‐500, the calcination temperatures to obtain intermediate Fe_3_O_4_/C are 400 and 500 °C, and other condition is same as the above‐mentioned hollow FeP/C nanosheets.

*Materials characterization*: X‐ray diffraction (XRD) patterns were collected using a Rigaku D/Max‐γB diffractometer with Cu *K*_α_ radiation. The morphology and structures of the products were characterized by using a field‐emission scanning electron microscope (FE‐SEM, FEI Quanta 200F), and a transmission electron microscope (TEM, JEOL JEM‐2100F) with an accelerating voltage of 200 kV. The thickness of the Fe‐glycolate nanosheets was determined by Bruker Dimension ICON‐Pt AFM. Raman spectra were taken under ambient conditions by using a micro‐Raman spectrometer (Reinshaw Raman Scope RM3000). Thermogravimetric analysis (TGA) was conducted on SDT Q600 (TA Instruments). Nitrogen adsorption‐desorption was performed on Autosorb 6B at –196 °C. XPS of the sample was conducted on a VG K_α_ Probe spectrometer (Thermo Fisher Scientific) with Al K_α_ radiation as the excitation source.

*Electrochemical measurements*: All the electrochemical catalytic measurements were performed in 0.5 m H_2_SO_4_ solution using a standard three‐electrode system (Wavedrive 20, PINE Research Instrumentation) with catalyst, saturated calomel electrode (SCE), and graphite rod acting as the working, reference, and counter electrode, respectively. A total of 5 mg of catalyst was dispersed into 470 µL of ethanol and 30 µL of Nafion (5 wt% in a mixture of alcohols and water, Sigma–Aldrich) and sonicated for 20 min. Then, 40 µL of homogeneous dispersion solution was drop‐cast onto the surface of the glassy carbon electrode at a catalyst loading of 1.6 mg cm^−2^. All of the potentials were calibrated with an RHE using the following equation: *E_RHE_* = *E_SCE_* + 0.26 V. In addition, the data were all corrected against iR loss. The durability for each electrocatalyst is performed by continuous linear sweep voltammetry scans from –0.2 to –0.6 V (vs SCE) with a sweep rate of 50 mV s^−1^. Electrochemical impedance spectroscopic (EIS) measurements were performed on CHI660E at a potential of 40 mV in the frequency range from 0.01 to 10^6^ Hz with a single modulated AC potential of 10 mV.

## Conflict of Interest

The authors declare no conflict of interest.

## Supporting information

Supplementary
