# Supplementary material for: Construction of FeP Hollow Nanoparticles Densely Encapsulated in Carbon Nanosheet Frameworks for Efficient and Durable Electrocatalytic Hydrogen Production
Source: Adv Sci (Weinh). 2018 Dec 11;6(3):1801490. doi: 10.1002/advs.201801490 (PMC6364707; doi:10.1002/advs.201801490)
Supplement: Supplementary file 1 — Supplementary [file ADVS-6-1801490-s001.pdf]

Copyright WILEY-VCH Verlag GmbH & Co. KGaA, 69469 Weinheim, Germany, 2018.

Supporting Information

for *Adv. Sci.,* DOI: 10.1002/advs.201801490

Construction of FeP Hollow Nanoparticles Densely Encapsulated in Carbon Nanosheet Frameworks for Efficient and Durable Electrocatalytic Hydrogen Production

Fei-Xiang Ma, Cheng-Yan Xu, Fucong Lyu, Bo Song, Shu-Chao Sun, Yang Yang Li, Jian Lu, and Liang Zhen**

Copyright WILEY-VCH Verlag GmbH & Co. KGaA, 69469 Weinheim, Germany, 2016.

Supporting Information

Construction of FeP Hollow Nanoparticles Densely Encapsulated in Carbon Nanosheet Frameworks for Efficient and Durable Electrocatalytic Hydrogen Production

Fei-Xiang Ma, Cheng-Yan Xu,* Fucong Lyu, Bo Song, Shu-Chao Sun, Yang Yang Li, Jian Lu, and Liang Zhen*

Dr. F.-X. Ma, Prof. C.-Y. Xu, S.-C. Sun, Prof. L. Zhen
State Key Laboratory of Advanced Welding and Joining
Harbin Institute of Technology
Harbin 150001, China
E-mail: cy_xu@hit.edu.cn, lzhen@hit.edu.cn
Dr. F.-X. Ma, Prof. C.-Y. Xu, S.-C. Sun, Prof. L. Zhen
MOE Key Laboratory of Micro-Systems and Micro-Structures Manufacturing
Harbin Institute of Technology
Harbin 150080, China
Dr. F.-X. Ma, F. Lyu, Prof. J. Lu
Department of Mechanical Engineering
City University of Hong Kong
Kowloon, Hong Kong, China
Prof. B. Song
Centre for Composite Materials and Structures
Harbin Institute of Technology
Harbin 150080, China
Prof. Y. Y. Li
Department of Materials Science and Engineering
City University of Hong Kong
Kowloon, Hong Kong, China

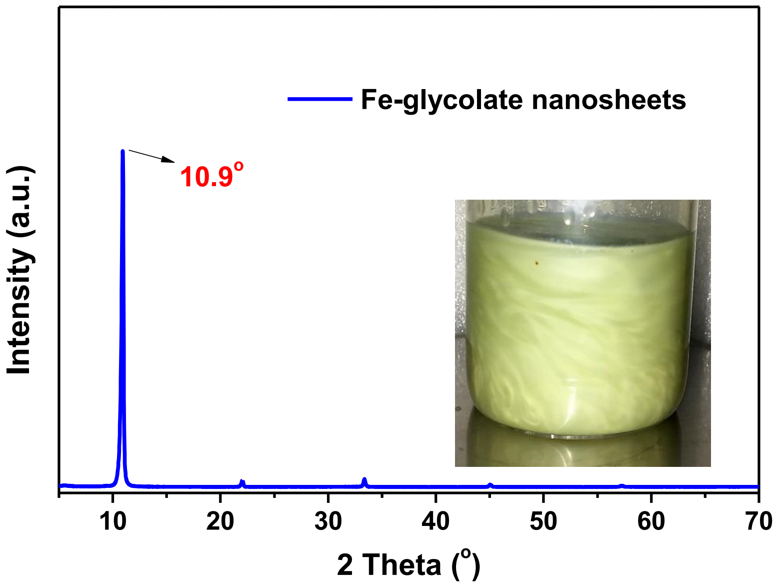

Figure S1 XRD pattern of Fe-glycolate nanosheet precursor. Inset is the digital photograph of Fe-glycoate precursor product in ethanol dispersion.

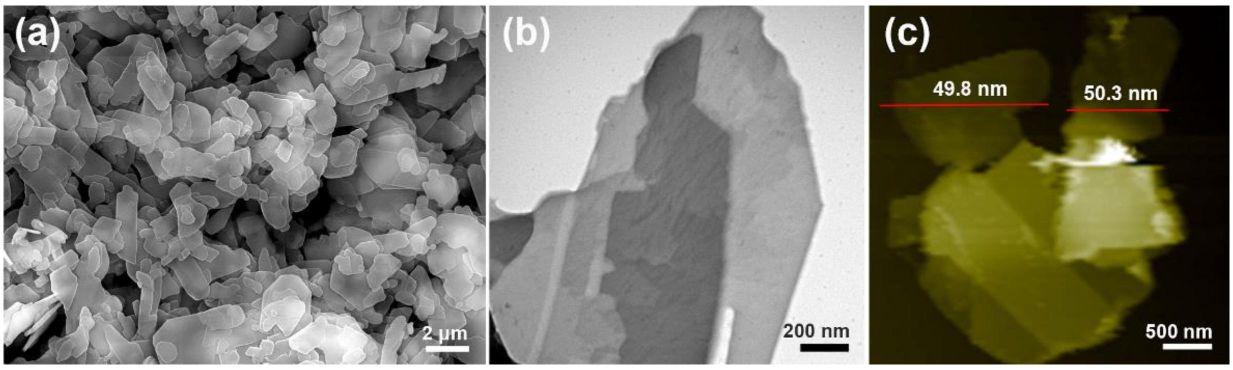

Figure S2 (a) SEM, (b) TEM and (c) AFM images of Fe-glycolate precursor nanosheets. (red lines in (c) reveal the thickness of Fe-glycolate is about 50 nm)

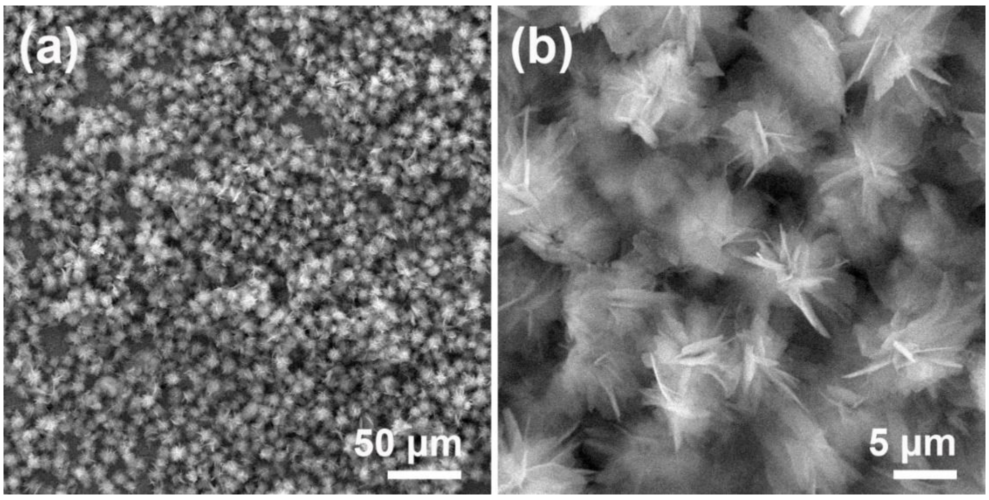

Figure S3 SEM images with different magnifications of Fe-glycolate nanosheet precursor with a short reaction duration of 30 min. The three-dimensional hierarchical structures are dissociated into well-dispersed Fe-glycolate nanosheets after increasing the reaction time to 2 h.

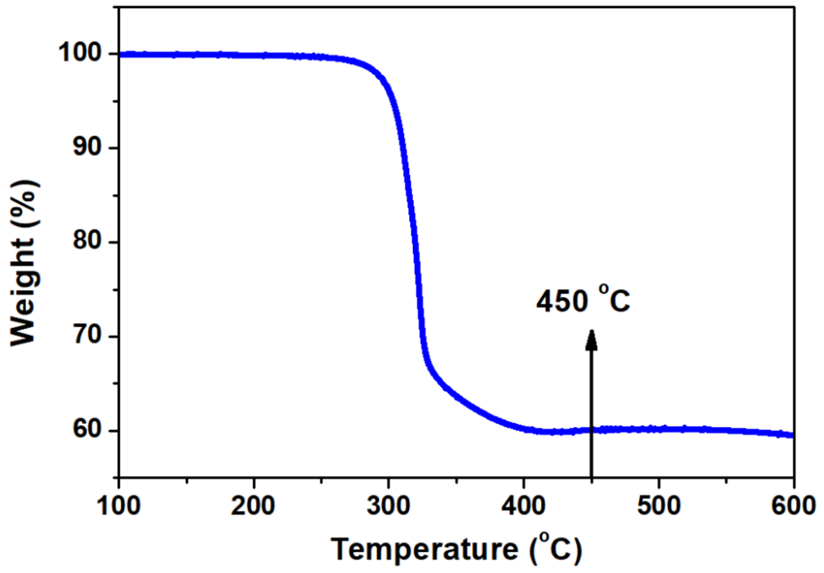

Figure S4 TGA curve of Fe-glycolate precursor nanosheets.

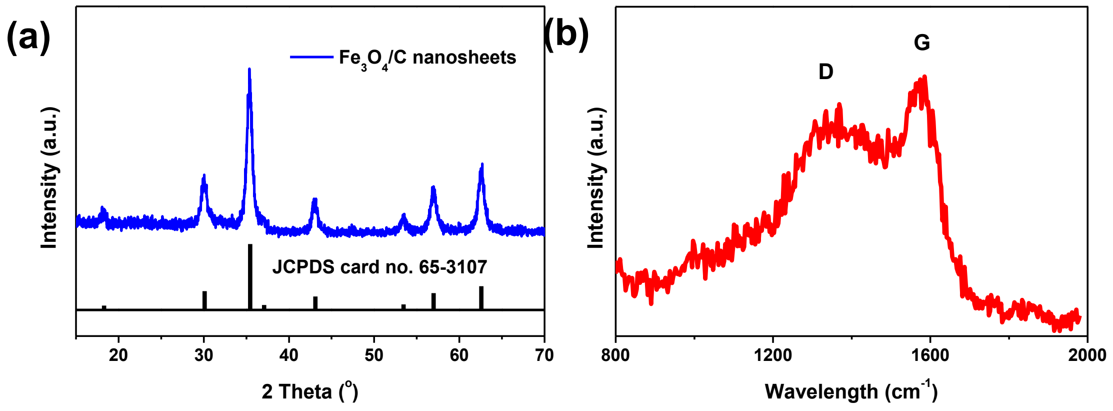

Figure S5 (a) XRD pattern and (b) Raman spectra of as-calcined Fe_3O_4/C nanosheets.

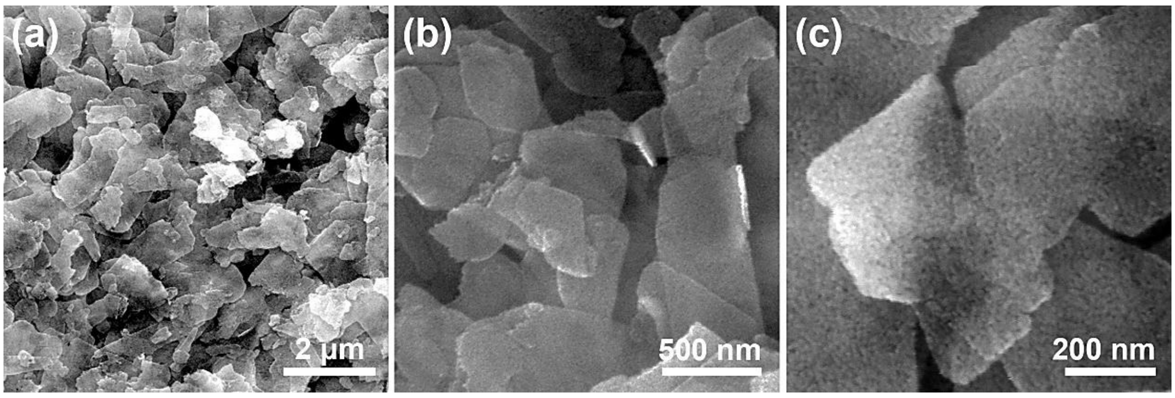

Figure S6 SEM images with different magnifications of as-calcined Fe_3O_4/C nanosheets.

The 2D Morphologies of Fe-glycolate precursor nanosheets can be perfectly maintained after calcination at 450 ^{o}C for 2h. At high magnification (Figure S6c), small nanoparticles can be clearly observed.

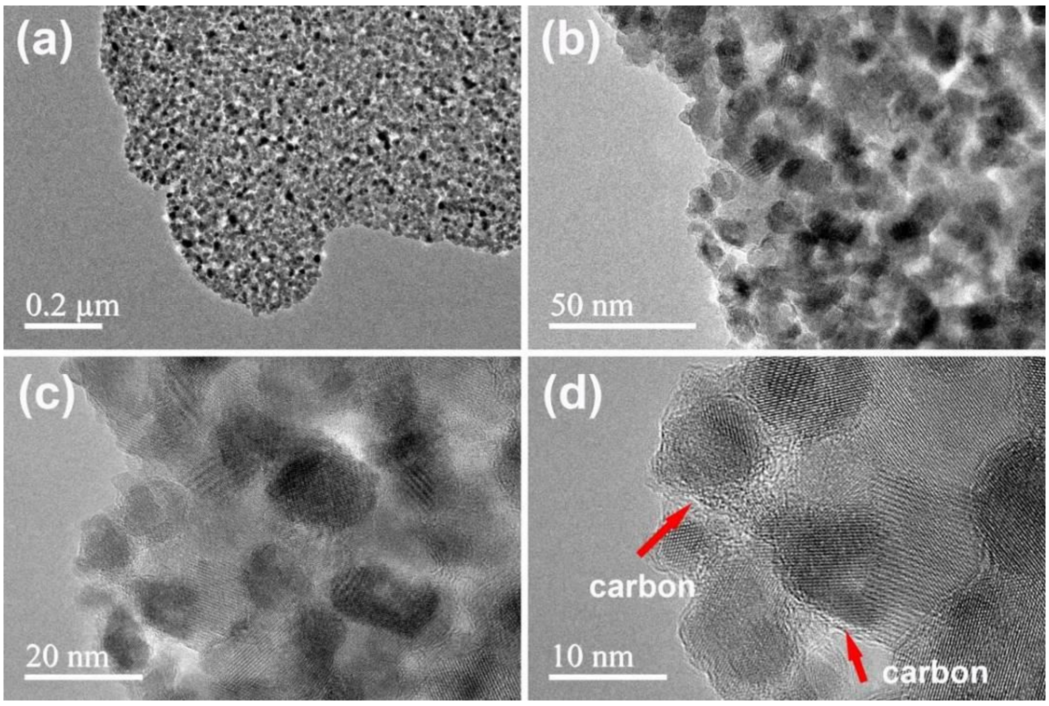

Figure S7 (a-c) TEM images with different magnifications and (d) HRTEM image of as-calcined Fe_3O_4/C nanosheets. The red arrows in (d) show amorphous carbon in the surface of Fe_3O_4 nanoparticles

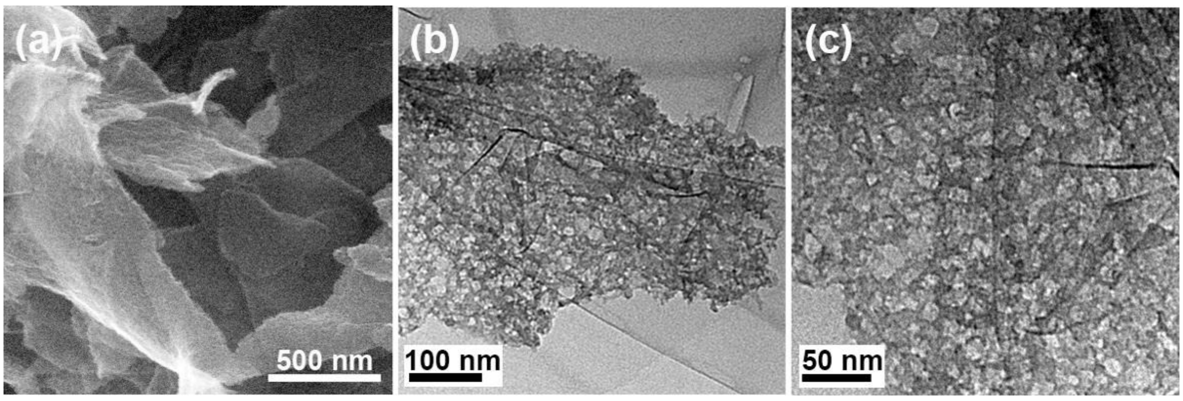

Figure S8 (a) SEM and (b, c) TEM images of as-calcined carbon nanosheets after selective removing Fe_3O_4 nanoparticles from Fe_3O_4/C using 4 M HCl solution.

As can be seen, the nanosheet feature can be still reserved from the SEM image (Figure S8a). Through the TEM results, numerous pores, produced by the removed Fe_3O_4 nanoparticles, are distributed in the carbon nanosheet frameworks. Therefore, it is reasonable to conclude that the Fe_3O_4 nanoparticles are embedded in the carbon nanosheet frameworks.

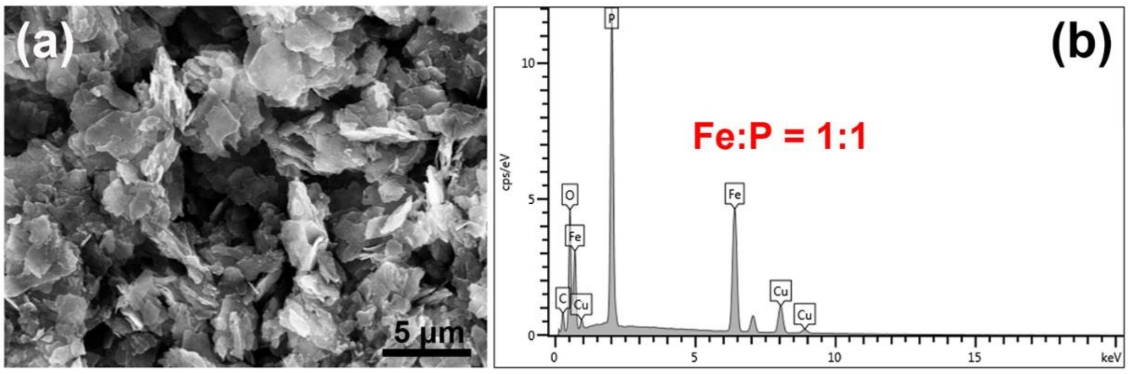

Figure S9 SEM image (a) and corresponding EDS result (b) of hollow FeP/C nanosheets.

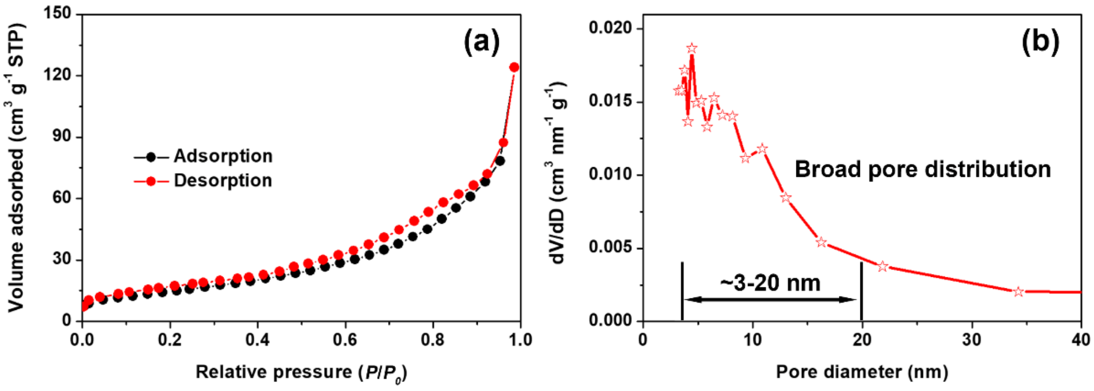

Figure S10 Nitrogen adsorption-desorption isotherm (a) and the corresponding pore size distribution (b) curves of as-prepared FeP/C-450 hollow nanosheets.

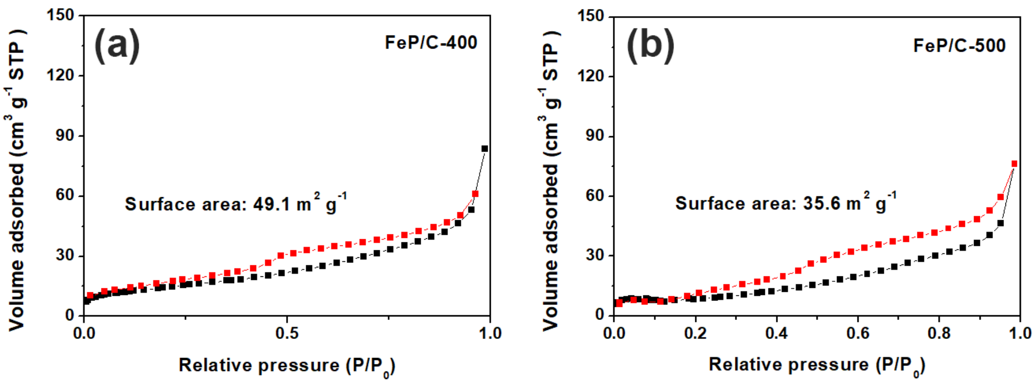

Figure S11 Nitrogen adsorption-desorption isotherm of as-prepared FeP/C hollow nanosheets calcined at different temperatures. (a) 400 ^{o}C; (b) 500 ^{o}C.

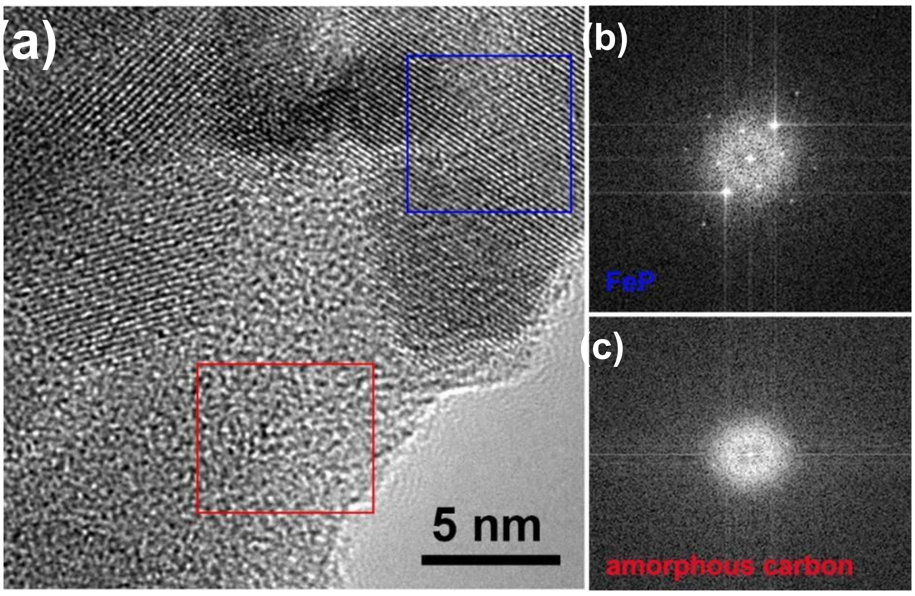

Figure S12 HRTEM image (a) and the corresponding FFT patterns (b, c) of the H-FeP/C nanosheets.

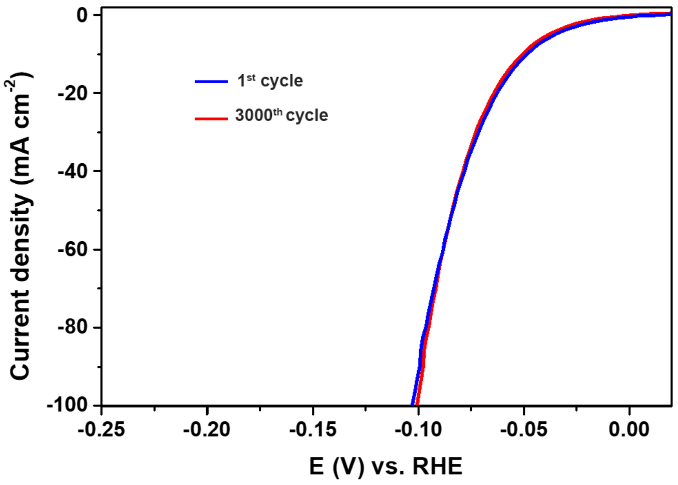

Figure S13 Polarization curves of as-prepared FeP/C-450 after continuous potential sweeps at 50 mV s^{-1}.

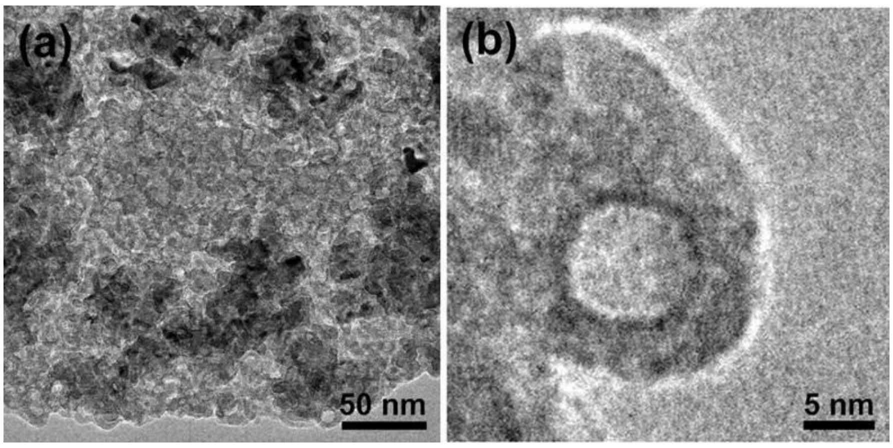

Figure S14 TEM images of FeP/C-450 hollow nanosheets after continuous potential sweeps.

As can been seen, the overall 2D morphology of FeP/C nanosheets can be well-preserved, and the hollow FeP nanoparticles can be also survived in the harsh operating condition.

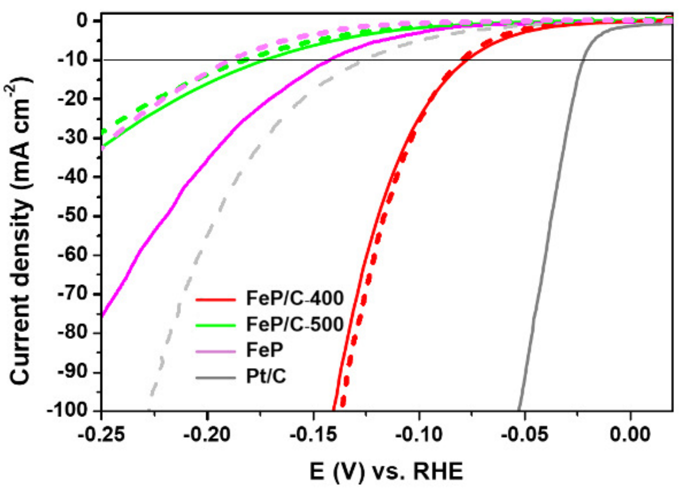

Figure 15 Polarization curves of other electrocatalysts including FeP/C-400, FeP/C-500, FeP and Pt/C after continuous potential sweeps at 50 mV s^{-1}.

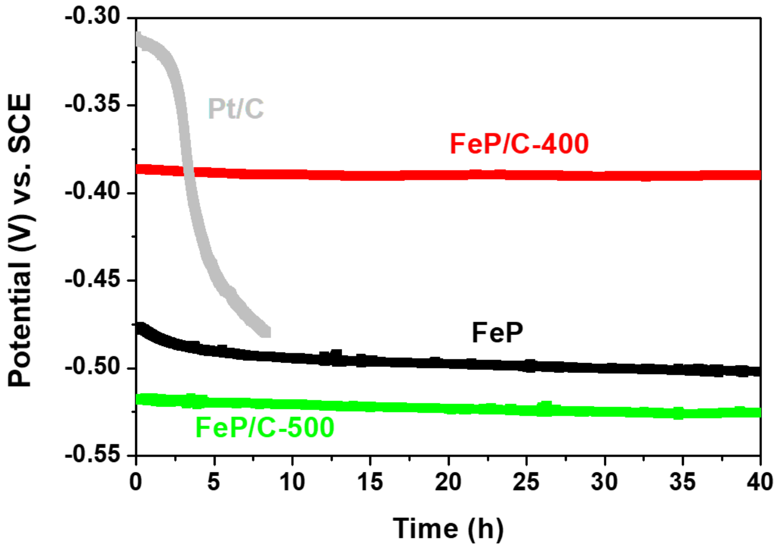

Figure S16 Potential change of other electrocatalysts (FeP/C-400, FeP/C-500, FeP and Pt/C) at a constant current density of 20 mA cm^{-2}.

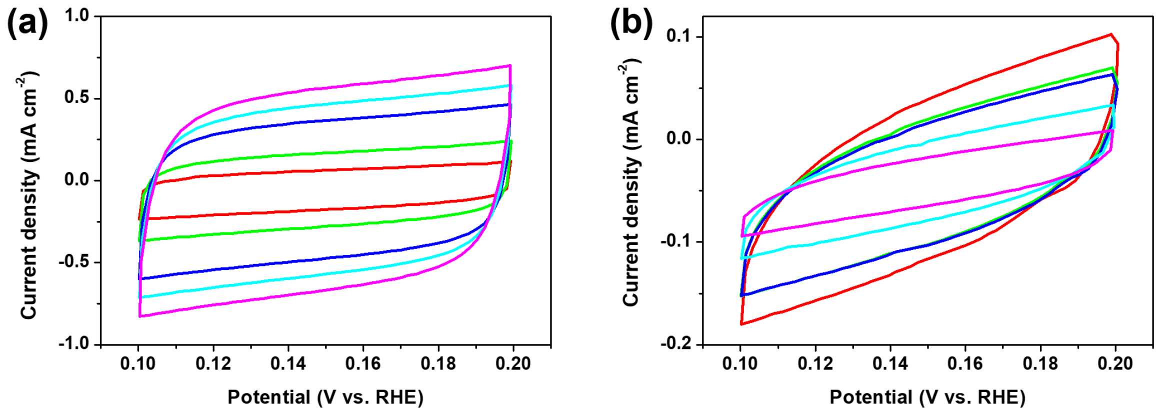

Figure S17 Cyclic voltammograms for (a) FeP/C-400 and (b) FeP/C-500 with different scan rates from 10 to 60 mV s^{-1} in the potential range of 0.1–0.2 V versus RHE.

Table S1 Comparison of HER performance in acidic media of the FeP/C-450 hollow nanosheets with representative non-Pt HER electrocatalysts.

**Electrocatalysts**	**Overpotential (mV) @10 mA cm^{-2}**	**Tafel slope (mV dec^{-1})**	**Refs.**
FeP nanoparticles coated by carbon	71	52	*J. Am. Chem. Soc.* **2017**,139, 6669
FeP@C derived from polymer	52	49	*Adv. Funct. Mater.* **2015**, 25, 3899
Fe@FeP/CNTs	53	55	*Nano Lett.* **2017**, 17, 2057
FeP/RGO	150	65	*Chem. Asian. J.* **2018**, 13, 679
FeP nanorods/rGO	107	58.5	*Adv. Sci.* **2015**, 2, 1500120
Fe_xP@N doped Carbon	227	81	*Nano Energy* **2017**, 35, 115
Carbon coated hollow FeP microcubes	115	56	*J. Mater. Chem. A* **2016**, 4, 8974
FeP@C	130	67	*Nanoscale* **2017**, 9, 3555
Mo_2C nanotubes	172	62	*Angew. Chem. Int. Ed.* **2015**, 54, 15395
NiP	~115	46	*J. Am. Chem. Soc.* **2013**, 135, 9267
CoP nanoparticles	~72	50	*Angew. Chem. Int. Ed.* **2014**, 53, 5427

MoP/CNTs	83	60	*Adv. Funct. Mater.* **2018**, 28, 1706523
CoS_2/MoS_2/RGO	98	37.4	*Adv. Funct. Mater.* **2017**, 27, 1602699
MoO_2@PC-RGO	64	41	*Angew. Chem. Int. Ed.* **2015**, 54, 12928
FeP nanorods/Ti plate	70mV (Intergrated electrode)	39	*Nanotechnology,* **2017**, 28, 105705
FeP nanorods supported by graphene	53 mV (Intergrated electrode)	42	*J. Mater. Chem. A,* **2017**, 5, 11301
FeP nanorods on a carbon fiber paper	31 mV (Intergrated electrode)	53	*J. Mater. Chem. A*, **2016**, 4, 1454
Rague-Like FeP nanocrystals/carbon cloth	34 mV (Intergrated electrode)	29.2	*Nanoscale*, **2015**, 7, 10974
Fe_2P/nitrogen-doped graphene hybrid	138 mV	67	*Nano Energy*, **2015**, 12, 666
FeP Nanoparticles/carbon cloth	~40 mV (Intergrated electrode)	32	*ACS Appl. Mater. Interfaces*, **2014**, 6, 20579
**FeP/C hollow nanosheets**	**51.1 mV**	**41.7**	**this work**
